# Proteomic analysis of the metabolic adaptation of the biocontrol agent *Pseudozyma flocculosa *leading to glycolipid production

**DOI:** 10.1186/1477-5956-8-7

**Published:** 2010-02-09

**Authors:** Walid Hammami, Florian Chain, Dominique Michaud, Richard R Bélanger

**Affiliations:** 1 Département de Phytologie, Centre de recherche en horticulture, Université Laval, Québec G1V 0A6, Canada

## Abstract

The yeast-like epiphytic fungus *Pseudozyma flocculosa* is known to antagonize powdery mildew fungi through proliferation on colonies presumably preceded by the release of an antifungal glycolipid (flocculosin). In culture conditions, *P. flocculosa *can be induced to produce or not flocculosin through manipulation of the culture medium nutrients. In order to characterize and understand the metabolic changes in *P. flocculosa *linked to glycolipid production, we conducted a 2-DE proteomic analysis and compared the proteomic profile of *P. flocculosa *growing under conditions favoring the development of the fungus (control) or conducive to flocculosin synthesis (stress). A large number of protein spots (771) were detected in protein extracts of the control treatment compared to only 435 matched protein spots in extracts of the stress cultures, which clearly suggests an important metabolic reorganization in slow-growing cells producing flocculosin. From the latter treatment, we were able to identify 21 protein spots that were either specific to the treatment or up-regulated significantly (2-fold increase). All of them were identified based on similarity between predicted ORF of the newly sequenced genome of *P. flocculosa *with *Ustilago maydis' *available annotated sequences. These proteins were associated with the carbon and fatty acid metabolism, and also with the filamentous change of the fungus leading to flocculosin production. This first look into the proteome of *P. flocculosa *suggests that flocculosin synthesis is elicited in response to specific stress or limiting conditions.

## Introduction

*Pseudozyma flocculosa *(Traquair, Shaw, and Jarvis) Boekhout and Traquair, a basidiomycetous yeast originally classified as *Sporothrix flocculosa*, is a powerful and specific antagonist of powdery mildew fungi whose mode of action appears to be unique among other related *Pseudozyma *species [1]. It is able to colonize powdery mildew colonies within a few hours following its contact with the pathogen and this ability was preceded and/or facilitated by the release of an antifungal glycolipid. This glycolipid, named flocculosin, was recently isolated and purified from solid cultures of *P. flocculosa *[2]. Extracellular glycolipids are produced by a large variety of microorganisms and may serve different purposes to confer fitness advantages. Interestingly, the structure of flocculosin is closely related to the cellobiose lipid, ustilagic acid, produced by the plant pathogen *Ustilago maydis *[2]. In a previous study, the absence of growth factors contained in yeast extract, combined with high carbon availability were described as stress conditions inducing the production of flocculosin in *P. flocculosa *cultures [3]. However, it is unclear how these conditions relate to the antagonistic behavior of the fungus in nature.

In spite of recent technical advances in molecular biology, the molecular basis of metabolic changes of *P. flocculosa *under stress condition is poorly understood because of the limited knowledge about the genetics of *Pseudozyma *spp. in general and of *P. flocculosa *in particular. Recently, Marchand *et al. *[4] isolated and studied the expression of a putative homolog of a *cyp1 *gene involved in the biosynthesis of ustilagic acid in *U. maydis *[5]. They showed a direct correlation between *cyp1 *expression and flocculosin production in stressed cultures of *P. flocculosa *but did not observe major differences in *cyp1 *expression when the fungus was developing on healthy or powdery-mildew infected leaves. These findings offered a relative appreciation of gene expression without information on the post-translational modifications or the relative concentration of the gene products (protein). Given that no strong correlation necessarily exists between the amount of a given protein and its transcripts [6], an in-depth analysis of the *P. flocculosa *proteome under comparative conditions would provide more precise information and a better understanding of the global cellular response of *P. flocculosa *that can trigger its biocontrol activity.

The most common implementation of proteome analysis is the separation of proteins by two-dimensional gel electrophoresis (2-DE) and their identification by peptide analysis with mass spectrometry (MS) analytical methods. Upon encountering new growth conditions, the microorganism under study will alter its protein synthesis in order to adapt to the new environment. Thus, by applying proteomic techniques such as 2-DE, different protein patterns from the same organism exposed to different environments can be compared. For example, Böhmer *et al. *[7] were able to study the dimorphic transition from budding to filamentous growth in *U. maydis *by comparing the proteome maps of the two forms by 2-DE. They thus identified 13 protein spots that were significantly enhanced during filamentous growth induced by the bW2/bE1-heterodimer. A similar approach was used to characterize virulence factors of *Botrytis cinerea *[8].

The objective of this study was to characterize the metabolic changes in *P. flocculosa *under two specific conditions inducing morphological characteristics conducive or not to flocculosin synthesis. For this purpose, we conducted a comparative analysis of the protein profile of each condition using a 2-DE approach. Taking advantage of the recent sequencing of the *P. flocculosa *genome, proteins of interest were matched with predicted ORFs in *P. flocculosa *and identified with a high level of confidence based on similarity to entries in available genome databanks including that of *U. maydis*.

## Materials and methods

### Fungal material

*P. flocculosa *(DAOM 196992) was used throughout this study. Stock cultures consisted of conidia lyophilized in maltose (20%) and kept at -80°C as aliquots of ca. 1 × 10^6 ^cells. Mother cultures were obtained by inoculating 100 ml of YMPD medium (yeast extract 3 g/l, malt extract 3 g/l, peptone water 5 g/l and dextrose 10 g/l) in a 500 ml baffled flask with one bottle of lyophilized cultures previously hydrated with 3 ml of sterile water. All culture media ingredients were supplied by Difco (BD Biosciences, Mississauga, Ontario, Canada). Seed cultures consisting of sporidia, were prepared by inoculating 100 ml of YMPD medium with 5 ml of a 3-day-old mother culture. The seed cultures were maintained on a rotary shaker set at 150 rpm at room temperature and were transferred into a fresh medium every three days for a maximum period of 30 days.

### Culture conditions

Two specific culture conditions for *P. flocculosa *as defined by Hammami *et al. *[3] were used in this study: one favoring morphological characteristics leading to sporidia production (control), and one favoring limited germination of the spores and mycelial fragments with associated production of flocculosin (stress). Briefly, 5 ml of the seed culture were used to inoculate 100 ml of YMPD_0.5 _medium (yeast extract 3 g/l, malt extract 3 g/l, peptone water 2.5 g/l and dextrose 5 g/l) in a 500-ml baffled flask. To stress the cells into producing flocculosin, a 10-ml sucrose solution (10%) (w/v) was added to the medium after 72 h, and this process was repeated 24 h later. For the control conditions, no amendments were made to the medium for the duration of the culture. After 120 h, the cell biomass was collected by filtration and used for protein extraction.

### Protein extraction

Fungal cells from both control and stress conditions were ground to a fine powder in liquid nitrogen using a cooled mortar. The extraction procedure consisted of protein solubilization with phosphate buffer followed by protein precipitation with trichloroacetic acid (TCA)/acetone (phosphate-TCA-acetone). The cell powder (4 g) was suspended in 10 ml of 10 mM potassium-phosphate buffer (pH 7.4) containing 0.07% w/v dithiothreitol (DTT) (Sigma-Aldrich, St. Louis, MO) and 200 μl of protease inhibitor cocktail for fungi and yeasts (Sigma, St. Louis, MO). The mixture was stirred at 4°C for 2 h and the extract clarified by centrifugation (15 min, 20 000 *g*). The supernatant was recovered and the proteins were precipitated by the addition of an equal volume of 10% w/v TCA in acetone containing 0.07% w/v DTT. The proteins were allowed to precipitate for 1 h at -20 C and the protein pellet recovered after centrifugation (15 min, 15 000 *g*) was washed twice with acetone containing 0.07% w/v DTT. The pellet was then solubilized in an electrophoretic sample buffer consisting of 8 M urea (Sigma-Aldrich) containing 2% (w/v) CHAPS, 0.5% (v/v) IPG buffer pH 3-10, and 60 mM DTT. Protein concentration in the samples was determined using the method of Bradford [9], with ovalbumin as standard protein according to the supplier's recommendation (Bio-Rad, Hercules, Ca). Final sample volumes of each extract were adjusted to normalize the amount of protein per sample to 300 μg/250 μl.

### 2-DE

For IEF, the samples were diluted in sample buffer containing a few grains of bromophenol blue, to a concentration of 400 μg/250 μl. The samples were then loaded on immobiline IPG strips (13 cm) with a linear pH 3-10 gradient, and resolved using an IPGphor apparatus (Amersham Biosciences, Baie d'Urfé, Québec,). After active rehydration for 12 h, IEF was performed following a voltage step-gradient (100 V for 1 h, 500 V for 1 h, 1000 V for 1 h, 5000 V for 1 h, and 8000 V to reach 47320 Vh). Before SDS-PAGE, the IPG strips were equilibrated for 15 min in a solution containing 6 M urea, 50 mM Tris-HCl pH 8.8, 2% (w/v) SDS, 30% (v/v) glycerol, and 60 mM DTT, and then equilibrated for 15 min in the same solution after substituting DTT with 5% (v/v) iodoacetamide (Sigma-Aldrich, St. Louis, MO). The second dimension was carried out on a 1 mm thick 12% (w/v) polyacrylamide gel. The gels were run at constant 30 mA until the bromophenol blue dye front migrated 2 cm from the bottom. The proteins were fixed overnight in water containing 10% (v/v) acetic acid, 50% (v/v) methanol on a rocking platform at low speed. The gels were finally rinsed three times in Milli-Q water and stained with the GelCode blue reagent (Pierce, Rockford, IL) following the manufacturer's instructions. Three replicate gels from individuals exposed to the same experimental conditions were performed to allow for subsequent statistical assessments.

### Image analysis and protein identification

#### *P. flocculosa *genome sequencing

Genomic DNA was extracted using the Qiagen DNeasy Plant Mini Kit from a 10-ml aliquot of a 3-day-old culture of *P. flocculosa*. Using 454 sequencing Titanium technology (454 Life Sciences Corp), the DNA genome of *P. flocculosa *was sequenced at the Genome Quebec Innovation Center (Montréal, QC, Canada). Briefly, 1.4 million reads were generated and 80% of them were fully assembled into 3410 contigs. Due to the large size of this genome (22 Mb), a long paired end sequencing approach was used to arrange the contigs (order/orientation) into 1281 scaffolds. The sequences contained on these scaffolds were used for protein identification based on matches with predicted ORFs and extraction of the corresponding sequences for further analysis (see below).

#### Protein identification

The 2-DE gels were digitized and analyzed using the Phoretix 2D Expression software, v. 2005 (NonLinear USA Inc., Durham, NC). Protein spot intensities were normalized using the 'total spot volume' method (i.e., each spot being expressed as a percentage of the total spot volume on that gel) to account for variations in emission levels between images, and a background subtraction was performed following the supplier's recommendations. Detection by gel to gel matching was performed to identify differences among treatments. All matched spots were detected on at least two gels from each set of three gels (replicates). Protein spots for identification were excised from the gels manually, and incubated for 15 min in 250 μl of water/acetonitrile (1:1 v/v). After additional washes in acetonitrile and 100 mM NH_4_-HCO_3_, the proteins were reduced with dithiothreitol, alkylated with iodoacetamide, and digested to peptides overnight at 37°C with MS grade Trypsin Gold (Promega Corporation, Madison WI, USA). Liquid chromatography (LC)-tandem MS was conducted at the Genome Quebec Innovation Centre of McGill University (Montréal QC, Canada), using a Q-time-of-flight (TOF) micro MS apparatus (Waters, Milford MA, USA) and a Nanosource modified with a nanospray adapter (New Objective, Woburn MA, USA) to hold a PicoFrit column (BioBasic C18 packing, 5 μm, 300 Å). The inferred peptides were searched against genomic data of the newly sequenced *P. flocculosa *database using the Basic Local Alignment Search Tool (BLAST) and the CLC Genomics Workbench software, v. 3.6.1 (CLC Bio USA, Cambridge MA, USA). Homologues to the predicted ORFs were searched in the National Center for Biotechnology Information (NCBI) database and the *Ustilago maydis *database of the Broad Institute http://www.broadinstitute.org/annotation/genome/ustilago_maydis/Home.html. Matched sequences in these databases were further analyzed against the *P. flocculosa *genome to ascertain that they referred back to the predicted ORFs.

## Results

### Culture conditions

**Figure 1:**
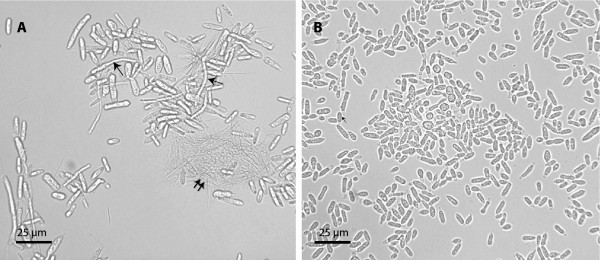
**Microscope photographs (400×) of *Pseudozyma flocculosa *(cell morphology and flocculosin production) in a 120-h old culture in YMPD medium amended (A) or not (B) with sucrose after 72 h**. Single arrows indicate elongating sporidia producing flocculosin; double-arrows indicate typical needle-shape crystals of flocculosin.

Protein extracts from *P. flocculosa *cells grown under two culture conditions were used for comparative purposes. Under control conditions, the conidial cells (sporidia) divided and maintained a yeast-like morphology without producing flocculosin (Fig. 1a). Under stress conditions, sporidia and mycelial fragments started elongating (pseudohyphal growth), a condition leading to flocculosin production as evidenced by the presence of needle-like crystals (Fig. 1b).

### Protein extraction and 2-DE

**Figure 2:**
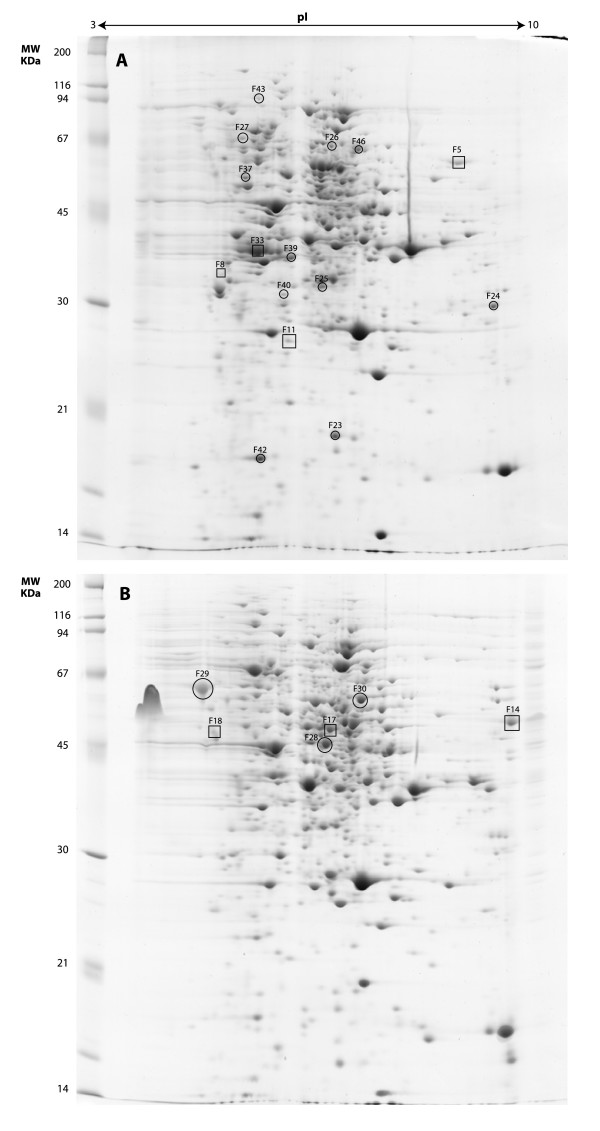
**2-D gels for (A) the proteome of *Pseudozyma flocculosa *under stress conditions leading to flocculosin production and (B) the proteome of *P. flocculosa *in yeast-like conditions (control; no flocculosin production)**. Circles indicate proteins that are modulated according to the culture medium; squares indicate proteins repressed or induced *de novo*.

**Figure 3:**
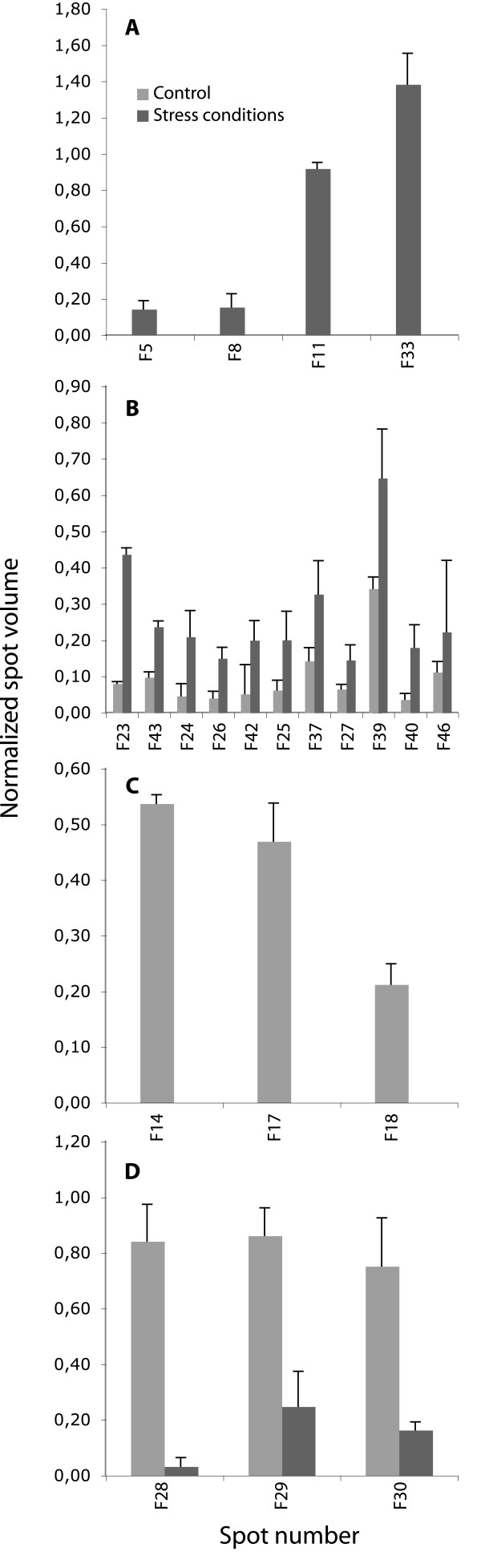
**Volume of selected proteins in extracts from *Pseudozyma flocculosa *cells grown under stress and control conditions**. Each of the 21 spots was analyzed in three 2-DE replicate gels. A) Proteins expressed *de novo *in stress conditions, B) Constitutive proteins up-regulated by at least two fold, C) Proteins completely repressed in stress conditions and D) Proteins down-regulated by at least two fold.

Total proteins were isolated from the cells of each culture and resolved by 2-DE (three 2-DE replicate gels for each condition) using IEF along a linear pH 3-10 gradient, followed by SDS-PAGE to resolve the proteins according to their molecular mass. In brief, a comparative analysis of the 2-D gels revealed striking qualitative and quantitative differences in protein levels between the two treatments. A large number of protein spots (771) were detected in protein extracts of the control treatment compared to only 435 matched protein spots in extracts of the stress conditions, which clearly suggests an important metabolic reorganization in germinating cells producing flocculosin. A number of spots were selected for identification to further characterize the proteome of *P. flocculosa *actively producing flocculosin. Twenty protein spots specific to [or up-regulated in] the flocculosin-producing treatment were first selected based on their unique presence in the stress treatment, or based on an important increase (= 2-fold, significant at *P *= 0.05) compared to control cells (Fig. 2). Ten additional spots were selected from control cells, based on criteria of uniqueness and high relative abundance (Fig. 2 and Fig. 3).

### Protein identification

Of the 30 spots selected and partially sequenced by LC-MS/MS (Table 1), 21 corresponded perfectly to a sequence within a predicted ORF of *P. flocculosa*. For all these 21 proteins, all short peptide sequences within the same protein used for analysis were consistently and uniquely associated with the same predicted ORF. In the case of the nine [9] unmatched proteins, the MS/MS data failed to generate the accurate peptidic sequences (non-significant score). Attempts to match those sequences in the *P. flocculosa *genome were unsuccessful.

**Table 1:** Comparative analysis of proteins regulated in a control culture medium or in a stress medium inducing flocculosin synthesis in *Pseudozyma flocculosa *cells.

Spot	Protein name	*GenBank *accession number	Mascot Score	Organism homology and respective *GenBank *accession number	Peptides sequences (LC MS/MS)
**Proteins specific to the stress treatment**					
F33	Thiamine biosynthesis	GQ922830	258	*Ustilago maydis* 71004594	K.VALLEPNDPSDVTEIIGSGK.V (2+) K.IGYVGEFGK.I (2+)
F5	Glutamate synthase	GQ922824	255	*Ustilago maydis* 71019533	K.GKDVIVIGGGDTGCDAQATALR.H (3+) R.LLMTNNFPEFTGR.V (2+) R.EADGVHFAMDFLAPNTK.S (3+) R.EYCISTTSFEK.D (2+) R.IGGLLMYGIPNMK.L (2+)
F8	Aspartic acid protease	157418069	96	*Pseudozyma flocculosa* 157418069	K.GTVYTDALTIGGLTASR.V (2+) K.LNAGVFQFTLAK.T (2+)
F11	24 kDa RAS-like protein	GQ922825	78	*Ustilago maydis* 71004934	R.NSFDEISTFHQQILR.V (3+) R.INVDEAFSNLVR.E (2+)
**Proteins up-regulated in the stress treatment**					
F23	Cyanate lyase	GQ922826	111	*Ustilago maydis* 71007509	R.LYEVLVVYGYPLK.H (2+) R.DPVLYR.L (2+)
F46	Transketolase	GQ922827	84	*Ustilago maydis* 39968757	K.IEAAIPELVGGSADLTGSNLTR.W (3+)
F24	Electron transfer flavoprotein beta subunit	GQ922828	201	*Ustilago maydis* 71004000	K.FSMNPFDEIAVEEAVR.L (2+)
F42	Triosephosphate isomerase	GQ922829	75	*Ustilago maydis* 164661864	K.VATAEQAQEVHAAIR.Q (3+)
F39	Transaldolase	GQ922831	268	*Ustilago maydis* 71020109	K.IASTWEGIQAAR.E (2+) K.LAEGIAGFAK.D (2+) K.TIVMGASFR.N (2+)
F40	Heat shock protein of HSP70 family	GQ922832	286	*Ustilago maydis* 71020701	R.LLGEFELTGIPPQPR.G (3+) R.VFTTVEDNQTQVTFPVYEGER.T (3+) R.KANITITNSVGR.L (3+) K.ANITITNSVGR.L (2+)
F27	Heat-shock protein 90*	GQ922823	177	*Cryptococcus neoformans* 25990448	K.GIVDSEDLPLNISR.E (2+) R.ELISNASDALDK.I (2+)
F37	ATP synthase beta chain	GQ922833	905	*Ustilago maydis* 71018215	R.IMNVIGEPMDER.G (2+) R.FTQAGSETSALLGR.I (3+) K.TVLIQELINNVAK.A(2+) K.VALVFGQMNEPPGAR.A(3+)
F25	Septin-8	GQ922834	180	*Ustilago maydis* 71019031	K.LTVIDTPGFGDYVNNR.D (2+) K.AGGHFTLMVVGESGVGK.T (3+) K.TVEIDIIK.A (2+)
F26	Succinate dehydrogenase [ubiquinone] flavoprotein subunit	GQ922835	232	*Ustilago maydis* 71005306	R.LGANSLLDIVVFGR.A (2+) R.TVIELEHFGLPFSR.T (3+) R.VMQSDAAVFR.T (2+)
F43	Alpha glucosidase precursor	GQ922836	116	*Ustilago maydis* 71016306	R.RDPDETLQPFFTLDAGTPVDSNMYGYHPVYTEAR.R (3+) R.RGLIQYR.A (2+)
**Proteins absent in the stress treatment**					
F14	Elongation factor 1-alpha	GQ922837	304	*Ustilago maydis* 71004810	K.IGGIGTVPVGR.V (2+) K.STTTGHLIYK.C (2+) R.EHALLAFTLGVR.Q 2(+) K.YYVTVIDAPGHR.D 3(+) K.SVEMHHEQLPEGLPGDNVGFNVK.N (3+)
F17	Ornithine amino-transferase	GQ922838	132	*Ustilago maydis* 71018171	K.GLLAKPTHVNIIR.L (3+) R.TGFGPFLDR.V (2+)
F18	6-phospho-gluconate dehydrogenase	GQ922839	99	*Ustilago maydis* 71014537	K.GILFVGSGVSGGEEGAR.H (2+) K.IVSYAQGFMLMR.E (2+)
**Proteins down-regulated in the stress treatment**					
F28	Phospho-glycerate kinase	GQ922840	280	*Ustilago maydis* 71021575	K.IQLIDNMLDK.V (2+) K.YSLKPVAAEVSK.L (2+) K.VNSLIICGGMAFTFK.K (3+) K.ALESPERPFLAILGGAK.V (3+)
F29	Phospho-glycerate kinase	GQ922840	62	*Ustilago maydis* 71021575	K.ALESPERPFLAILGGAK.V (2+) K.VNSLIICGGMAFTFK.K (2+) K.IQLIDNMLDK.V (2+)
F30	Glucose-6-phosphate 1-dehydrogenase	GQ922841	293	*Ustilago maydis* 71021693	K.LVDNVQITFK.E (2+) K.SFSAEDIRDEK.V (2+) K.DVTSGIFKDIPR.N (2+)

*Similarity was found in the MIPS *Ustilago maydis *database http://mips.helmholtz-muenchen.de/genre/proj/ustilago

When the full sequences of the 21 identified *P. flocculosa *ORFs were analyzed against the *U. maydis *and *NCBI *databanks, they all corresponded with the highest score to an annotated protein of *U. maydis *(Table 1). A reverse comparison of the 21 matching *U. maydis *proteins against the *P. flocculosa *genome confirmed the specific and unique association of these proteins with the respective ORF used for the initial analysis.

In all instances, the expression pattern of the identified proteins in *P. flocculosa *was fairly constant from one experiment to the next (Fig. 3). In general, our identifications revealed that most proteins induced *de novo *or up-regulated in the stress treatment were part of carbon (e.g. thiamine biosynthesis, transketolase), fatty acid (e.g. electron transfer flavoprotein) and biogenesis (e.g. Ras-like, septin) metabolic pathways. By comparison, proteins specific to [or more abundant in] control cells (e.g. elongation factor alpha, ornithine aminotransferase) were associated essentially with primary metabolic functions such as protein biosynthesis and amino acid metabolism. Spots F28 and F29 that were specific to the control treatment, were identified as being the same protein (phosphoglycerate kinase) in spite of their distinct migration behaviour (Fig. 2).

## Discussion

In this work, we have generated a first proteomic map of *Pseudozyma flocculosa*, a biocontrol fungus known for its antagonistic activity against powdery mildew fungi mediated, at least in part, by the release of an antifungal glycolipid, flocculosin. Our aim was to compare the proteomic profile of cell extracts grown under two specific morphological conditions, including one conducive to flocculosin synthesis, in order to gain a better understanding of the factors that could trigger the biocontrol activity of *P. flocculosa*.

Our results highlighted important qualitative and quantitative changes in the synthesis of individual proteins during the fungus' adaptation to a carbon-supplemented medium leading to flocculosin synthesis. As a first observation, we noted a clear decrease in the total number of proteins in the stressed cells, compared to control cells. This is indicative of an important reduction in certain metabolic activities that would be linked with the release of stress metabolites, namely flocculosin. Incidentally, Mimee *et al. *[10] have recently proposed that flocculosin could serve both roles of niche protection and food reserve under limiting conditions. This difference in metabolic behaviour was also accompanied with a gradual transition from the yeast-like to the filamentous form (see Fig. 1), the latter only being apt to produce flocculosin. While this transition step is not accompanied by an increase in biomass, as would be expected in typical filamentous fungi such as molds, Hammami *et al. *[3] suggested that it was induced as an adaptation/protection stage to a new environment for the epiphytic fungus. In line with these observations, some proteins associated with flocculosin biosynthesis in *P. flocculosa *were detected specifically in cells producing the glycolipid. For instance, a septin-like protein was recorded exclusively in cells grown in the carbon amended medium. Septins are GTPases that form filaments in fungi and animals, and are thought to function in controlling cytokinesis and coordinating nuclear division and membrane movement [11,12]. Together with septin, we also detected a Ras-like protein reported to promote filamentous growth in *U. maydis *[13].

Given that flocculosin synthesis was supported by C-fed-batch, it is also normal that several protein activities found in this treatment were linked to carbon metabolism. The expression levels of transketolase and transaldolase, for instance were 2 and 2.5 fold higher, respectively in the flocculosin-inducing medium. These two enzymes are involved in the non-oxidative part of the pentose phosphate pathway. The pentose phosphate pathway involves two steps: the first one (oxidative part) metabolizes glucose-6-phosphate to ribulose-5-phosphate, the principal carbon source for nucleotide synthesis and NADPH for reductive reactions in biosynthesis and perhaps for hydroxylation [14]. When the cell is not in suitable conditions to produce biomass, ribulose-5-phosphate joins the second step of the pathway (non-oxidative part), to be converted to glyceraldehyde-3-phosphate and two fructose-6-phosphate. The detection of thiamine biosynthesis activity in the flocculosin inducing medium is consistent with an increase in transketolase levels. Thiamine contributes to carbon metabolism by acting as cofactor of several enzymes such as pyruvate dehydrogenase, α-ketoglutarate dehydrogenase, branched-chain α-ketoacid dehydrogenase, transketolase and pyruvate decarboxylase [15]. Based on this, we can speculate that *P. flocculosa *does undergo a metabolic switch from biomass production to flocculosin production under stress conditions as proposed by Hammami *et al. *[3]. Indeed, the latter study found that the presence of yeast extract supported the conidial biomass production of *P. flocculosa*. When growth is blocked by the exhaustion of yeast extract, flocculosin excretion might constitute an overflow metabolism for *P. flocculosa *that regulates the intracellular energy level.

In addition to the observation of proteins consistent with the inhibition of biomass production under stress conditions, several other proteins were induced or increased in *P. flocculosa *cells following the addition of a carbon source in the medium. Higher levels of glutamate synthase, cyanate lyase and heat shock protein (HSP) 70 and 90 support the hypothesis that *P. flocculosa *was under stress conditions following the fed-batch. The addition of carbon increases the consumption speed of the components of the complex medium YMPD including the yeast extract ingredients. The exhaustion of these components, particularly yeast extract, appears to induce flocculosin synthesis, a strategy that may have been developed by *P. flocculosa *to store excess carbon in the environment as proposed by Mimee *et al. *[10]. As explained earlier, the carbon backbone of flocculosin (and of ustilagic acid) is composed of a long fatty acid chain originating from palmitic acid. Here, we detected the electron transfer flavoprotein, a key cofactor of the β-oxidation which is considered a way to produce acetyl-COA in the mitochondria. Equally, the expression of pentose phosphate-related enzymes is necessary to produce NADPH, which in turn is essential to fatty acid synthesis. Because of the complexity of the flocculosin (glycolipid) structure, its synthesis requires ATP production. Expectedly, an ATPase and other proteins such as the glycolytic enzyme, triose phosphate isomerase (TIM), succinate dehydrogenase and alpha-glucosidase involved in carbon metabolism producing the ATP molecule, were up-regulated by the carbon addition.

In the control medium, the cells tended to produce proteins that are commonly associated with biomass production. Ornithine aminotransferase has a fundamental role in the central metabolism of organisms, as it may serve for the synthesis of proline, polyamines, glutamate and glutamine. These products are of great nutritional and physiological importance for diverse functions including growth and development [16]. Also, the soluble elongation factors are likely to play an important role in limiting the error frequency during protein synthesis [17]. The low abundance of these proteins in the stress treatment suggests that carbon addition in the growth medium inhibited some activities which assure the appropriate course of cellular growth and shifted the overall metabolism toward the production of flocculosin.

Owing to the structural similarity between flocculosin and ustilagic acid, and to the phylogenetic link between *P. flocculosa *and *U. maydis*, we expected to detect in the flocculosin production treatment proteins belonging to the cluster of 12 co-regulated genes involved in ustilagic acid synthesis in *U. maydis *[18]. Our analyses did not identify any of the proteins related to this cluster of genes, whether they are present in the cytosol or in the membranes. The detection of membrane proteins such as the ATP-synthase seem to rule out the possibility that our extraction procedure failed to detect such proteins [19]. Their low relative concentration or a poor correlation between mRNA transcripts and protein accumulation for these 12 genes, as noted for many genes in yeast [6], could explain our results. A similar conclusion was reported in the study of the proteomic changes involved in the transition of *U. maydis *from budding to filamentous form [7]. Two proteins, versicolorin B synthase (UM03246) and anthranilate synthase (UM02376) that were minor components of multiprotein spots showed an increase in transcriptional level of 4- and 13-fold respectively.

Based on our observations, it does appear that *P. flocculosa *will only produce flocculosin early in the process of pseudohyphal formation under conditions of nutrient limitations. While this process can be artificially maintained in vitro by the addition of a C source, in nature it would serve as a means of protecting/forging an ecological niche as sporidia land in a new environment. This supports recent quantitative evaluation of flocculosin production *in situ *where Marchand *et al. *[4] found that genes involved in flocculosin synthesis were only up-regulated within the first 6 hours of sporidia coming into contact with a leaf. In the same manner, we can speculate that sporidia of *U. maydis *would produce ustilagic acid in the first steps of germination to facilitate contact with a compatible mating type, as suggested by Hewald *et al. *[5].

## Conclusion

This work presents the first proteomic map of *P. flocculosa *together with a positive identification of the salient proteins. The impressive number of proteins that displayed changes in their relative rates of synthesis during the adaptation to stress conditions conveys the idea that *P. flocculosa *is an organism in which gene expression varies in response to environmental changes leading to flocculosin synthesis. This analysis was greatly validated by the recent sequencing of *P. flocculosa *genome which allowed a perfect match of the peptide sequences of 21 proteins with specific predicted ORFs in *P. flocculosa*. Because of the close genetic link between *U. maydis *and *P. flocculosa*, we were able to positively link those proteins with a known function annotated in *U. maydis *thereby confirming the high level of homology and conserved genes between the biocontrol agent and the plant pathogen.

## Competing interests

The authors declare that they have no competing interests.

## Authors' contributions

WH, FC and RRB have made substantial contributions to the design of the experiments, data acquisition and interpretation. WH, DM and RRB have been involved in writing the manuscript and revising it critically for content. All authors have read and approved the final manuscript.
